# A systematic review on the dual roles of microRNAs in ischemic stroke: mechanisms and effects

**DOI:** 10.7717/peerj.21571

**Published:** 2026-07-27

**Authors:** Nur Iffah Ishak, Rosfaiizah Siran, Wan Nor I’zzah Wan Mohamad Zain, Nasibah Azme

**Affiliations:** 1 Multidisciplinary Initiative in Neuroscience Discovery and Science (MINDS), Faculty of Medicine, Universiti Teknologi MARA, Sungai Buloh, Selangor, Malaysia; 2 Laboratory Animal Care Unit (LACU), Faculty of Medicine, Universiti Teknologi MARA, Sungai Buloh, Selangor, Malaysia; 3 Department of Physiology, Faculty of Medicine, Universiti Teknologi MARA, Sungai Buloh, Selangor, Malaysia; 4 Department of Biochemistry and Molecular Medicine, Faculty of Medicine, Universiti Teknologi MARA, Sungai Buloh, Selangor, Malaysia; 5 Department of Medical Education, Faculty of Medicine, Universiti Teknologi MARA, Sungai Buloh, Selangor, Malaysia

**Keywords:** Ischemic stroke, MicroRNA, Neuroprotection, Mechanism, Effect

## Abstract

**Background:**

MicroRNAs (miRNAs) regulate key pathways in ischemic stroke with both protective and detrimental effects. Understanding these dual roles is important to developing novel therapeutic strategies for cerebral ischemia. This systematic review highlights key miRNAs under investigation, the dysregulation of miRNA expression, and the overall mechanism of miRNA function after ischemic stroke.

**Methods:**

A total of 20 preclinical *in vivo* studies from multiple databases (PubMed, Scopus, and Web of Science (WoS)) that modulate miRNA expression post-stroke, published within the last 5 years, were analyzed. To confirm its effectiveness, studies must include miRNA expression level, neurological function test, and infarction volume. Due to the heterogeneity of these studies, as evaluated by the SYRCLE risk of bias tool, a descriptive synthesis was employed to summarize the findings. This review was registered with PROSPERO (CRD42024552558).

**Results:**

During the onset of ischemia, there is a sudden upregulation of detrimental miRNAs, accompanied by downregulation of beneficial neuroprotective miRNAs. Beneficial miRNAs inhibited apoptosis, suppressed inflammation, reduced oxidative damage, protected the blood-brain barrier (BBB), promoted angiogenesis, and stimulated neurogenesis. Detrimental miRNAs inhibited critical neuroprotective signaling pathways, suppressed cellular defense mechanisms, promoted Reactive oxygen species (ROS) production, and accelerated ischemic injury. Some miRNAs play dual roles, promoting both apoptosis and neurogenesis.

**Discussion:**

MiRNA modulation demonstrated greater therapeutic potential when used in rationally designed combination therapies, such as two beneficial miRNAs (*e.g*., miR-210 + miR-126) or a combination of miRNA with other agents (*e.g*., miR-199a-5p inhibitor + AKT activator IGF-1). Despite the enormous potential of miRNA modulation therapy and numerous preclinical data, translating it from bench to bedside remains a challenge.

**Conclusion:**

MiRNAs play dual roles in ischemic stroke, mediating both injury and repair. Their pleiotropic actions position them as promising multi-targeted candidates for stroke therapy, provided translational barriers can be overcome.

## Introduction

Ischemic stroke, the most prevalent subtype of stroke, remains the leading cause of mortality and long-term disability worldwide (Feigin et al., 2025). This brain vascular disease arises from sudden obstruction of cerebral blood flow, which eventually triggers oxidative stress, systemic inflammatory responses, and neuronal apoptosis (Salaudeen et al., 2024). The narrow therapeutic windows and insufficient neuroprotection remain the main challenges in acute stroke interventions despite the advancements in reperfusion therapies, including thrombolysis and mechanical thrombectomy (Pérez-Mato et al., 2024). A new therapeutic option to limit secondary ischemic injury and promote recovery is critically important.

MicroRNAs (miRNAs) are small non-coding RNA molecules that regulate gene expression post-transcriptionally. MiRNAs are approximately 22 nucleotides in length and bind to complementary sequences in the 3′ untranslated region (3′UTR) of target messenger RNAs to typically repress translation and/or destabilize target mRNAs (Bartel, 2018). The miRNAs have been highlighted in various stroke pathophysiology processes as pivotal modulators, thus serving as potential targets for therapeutic intervention (Todoran et al., 2023). Studies have accumulated preclinical evidence demonstrating the regulation of miRNAs in neuroinflammation, neurogenesis, apoptosis, autophagy, and angiogenesis following stroke (Li et al., 2018). Mechanistically, these miRNAs exert their protective roles through complex regulatory networks.

The biological roles of miRNAs in ischemic stroke should be discussed under certain circumstances due to their complexity. Some miRNAs exhibit extensive neuroprotective properties and are promising targets for therapeutic intervention, while other miRNAs are consistently detrimental and represent intriguing targets for suppression. *In vitro* studies have demonstrated that the activation of miRNAs such as miR-21, miR-99a, and miR-497 acts to lessen the ischemic infarction volume and reduce apoptosis in neuronal cells (Buller et al., 2010; Tao et al., 2015; Yin et al., 2010) while the overexpression of miR-424 and miR-let-7c-5p suppresses microglia activation (Ni et al., 2015; Zhao et al., 2013). Interestingly, certain miRNAs have been demonstrated to exhibit both protective and harmful properties. MiR-181a plays a role in preventing inflammatory responses in macrophages in an *in vitro* model; however, an *in vivo* study demonstrated that miR-181a inhibition decreases neuronal apoptosis in forebrain ischemia (Moon, Xu & Giffard, 2013; Xie et al., 2013). Recent research also shows that miRNAs have a biphasic function. In early ischemia, MiR-335 suppresses HIF-1α and reduces brain infarction, but downregulation of MiR-335 promotes repair pathways during late post-stroke (Liu et al., 2015). MiRNAs can be profoundly neuroprotective in one context yet clearly harmful in another. This dual role of miRNA is due to their pleiotropic targets, cell-type specificity, and time-dependent expression after stroke (Branyan et al., 2022; Liu et al., 2013a; Xiang et al., 2019).

Research on miRNAs in ischemic stroke has advanced from monitoring individual miRNA alterations in human patients to identifying their direct targets and pathways in cell-based laboratory models. Finally, the proposed mechanisms are validated in *in vivo* models by inducing brain injury and assessing recovery. The most common method to induce cerebral ischemia in animals, particularly rodents, is the middle cerebral artery occlusion (MCAO) technique (Longa et al., 1989). This model, either transient (tMCAO) or permanent (pMCAO), consistently produces a focused cortical-striatal infarct that resembles the territorial damage observed in human stroke (Engel et al., 2011). Other less frequently adopted models include stereotaxic endothelin-1 injection for focal vasoconstriction, photothrombotic laser activation of a photosensitive dye, embolic clot of the internal carotid artery, global carotid artery occlusion, or direct electrocoagulation of the middle cerebral artery (Traystman, 2003; Zeng et al., 2023). The models allow researchers to investigate region-specific vulnerability, measure miRNA expression, identify cellular targets, and evaluate functional outcomes resulting from the modulation of specific miRNAs in ischemic situations.

Animal models of ischemic stroke serve as a central translational platform for miRNA research (Eyileten et al., 2025). Researchers have modulated miRNA expression in ischemic stroke animals by administering specific modulators, either as pre-treatment or post-treatment. Drugs administered before stroke induction provide neuroprotection against ischemic injury by stimulating endogenous defense mechanisms or inhibiting initial pathological cascades (Singh, Kharwar & Dandekar, 2022). However, pre-treatment has limited clinical translatability since ischemic stroke in humans often happens unexpectedly. On the other hand, drugs administered after stroke onset closely mimic clinical scenarios. A post-treatment miRNA therapy study assesses miRNA’s ability to reverse, stop, or lessen ongoing pathological processes, thereby enhancing recovery. Since ischemic stroke causes irreversible cellular damage and triggers inflammatory cascades, post-treatment efficacy may appear lower than pre-treatment in some studies (Bellut et al., 2023; Challa et al., 2022). However, this observation is likely influenced by factors such as the timing of interventions, the stage of disease progression, and experimental design.

This systematic review aims to comprehensively evaluate the dual roles of miRNAs in ischemic stroke, highlighting both their protective and detrimental effects and the mechanisms underlying these actions during disease progression and recovery. This review focuses on preclinical *in vivo* ischemic stroke models that modulate miRNA expression after ischemic stroke is induced. Numerous animal studies have been conducted; however, to the author’s knowledge, no clinical trials of miRNA modulation in ischemic stroke patients have been reported until today. By analyzing preclinical *in vivo* data from multiple databases and limiting the publications to the latest 5 years, this review highlights key miRNAs under investigation, their dual roles in influencing both protective and detrimental pathways, their implications for the therapeutic outcome of ischemic stroke, and the overall operating mechanism of miRNA after ischemic stroke attack.

## Materials and Methods

### Search strategy, inclusion and exclusion criteria

This systematic review was conducted in accordance with the Preferred Reporting Items for Systematic Reviews and Meta-Analyses (PRISMA) guidelines (Moher et al., 2009). Before the research began, the review protocol was registered with the International Prospective Register of Systematic Reviews (PROSPERO) under registration number CRD42024552558.

A comprehensive literature search was conducted across the PubMed, Scopus, and Web of Science (WoS) databases from their inception to 30 September 2025. The search strategy combined terms related to miRNAs and ischemic stroke using Boolean operators and appropriate Medical Subject Headings (MeSH) terms. The following keywords were used:

(“microRNA” OR “miRNA”) AND (“ischemic stroke” OR “cerebral ischemia” OR “cerebral infarction”) AND (“mechanism” OR “pathway” OR “signaling” OR “regulation”) AND (“effect” OR “role” OR “outcome” OR “function” OR “impact”) AND (“rat” OR “rats” OR “mouse” OR “mice” OR “rodent*” OR “rabbit*” OR “*in vivo”*).

The terms “ischemic stroke” and “cerebral infarction” were applied as MeSH terms in the PubMed database. The search was limited to peer-reviewed articles and original articles published in English only. The literature search was conducted from 2020 onward to capture the most recent advancements in miRNA research on ischemic stroke.

The eligibility criteria of screened studies were formulated based on Population, Intervention, Comparator, and Outcome (PICO) statements as follows:

1. Population/animals: Only animal models that have been experimentally induced with ischemic stroke are included for analysis.
2. Intervention/exposure: Eligible studies must involve an intervention that upregulates or downregulates miRNAs in animal models of ischemic stroke. The intervention must be administered either locally or systemically after stroke induction. The ischemic stroke model, time of treatment injection, frequency of intervention, and route of administration must be clearly reported.
3. Comparator/control: Only studies that compare miRNA-modulated groups to appropriate control groups are included.
4. Outcome: Included studies must provide all the following outcomes: quantitative data on the miRNA level that has been intervened, assessment of neurological function using standardized behavioral tests, and precise measurements of infarct size.
  Additional mechanistic outcomes, such as apoptosis, blood-brain barrier (BBB) disruption, and cytokine levels in brain tissue or serum, were also extracted where available.
5. Study design: Only preclinical *in vivo* studies, including randomized controlled trials and non-randomized controlled trials, were included.

Studies were excluded if they:

(i) involved human, *in vitro*, *ex vivo*, or *in silico* studies;
(ii) did not involve miRNA modulation as an intervention;
(iii) used miRNA pretreatment or genetically modified models (*e.g*., knockout/knockdown);
(iv) lacked sufficient details on miRNA intervention (*e.g*., timing, route, or frequency);
(v) did not include appropriate control groups;
(vi) did not report all required outcome measures;
(vii) were non-original articles (reviews, editorials, commentaries, protocols, conference abstracts, book chapters, or short communications);
(viii) were not published in English or lacked accessible full texts; or
(ix) provided insufficient or unclear data for interpretation.

It should be noted that studies were excluded when outcome data were insufficient for analysis. However, studies with adequate outcome reporting but incomplete methodological details, such as randomization, were retained and subsequently evaluated in the Risk of Bias assessment.

### Study selection and data extraction

The information on all eligible articles was downloaded and exported into Microsoft Excel. A rigorous multi-phase screening process has been established to ensure the inclusion of relevant, high-quality studies, based on predefined inclusion and exclusion criteria. The data management and duplicate removal were performed by two reviewers (NII, NA). Data were then screened and extracted. Initially, all articles were screened by three independent researchers (NII, NA, WNI) based on their titles. Any discrepancies were discussed and resolved through consensus. If consensus could not be reached, a fourth senior researcher (RS) mediated the decision. Articles that passed the title screening then underwent a title-and-abstract screening phase. The last evaluation phase was the full-text screening. Various websites were approached to retrieve full-text articles, including all Universiti Teknologi MARA’s (UiTM) subscribed digital databases. In every phase, three independent reviewers assessed each article, and any disagreements were arbitrated by a fourth reviewer. This structured multi-reviewer approach ensures a thorough selection process and minimizes bias. All decisions, including reasons for exclusion at each stage, will be documented to ensure transparency and reproducibility. Next (NII) and (NA) were assigned to extract data items from the selected articles, which include study characteristics and key findings. For studies involving multiple miRNA interventions, each intervention and its corresponding outcomes were extracted and reported separately to ensure clarity and avoid bias from data aggregation. The extracted data were then carefully examined by all reviewers.

### Risk of bias assessment

The quality of included studies was evaluated by two independent reviewers (NII, NA) using the SYRCLE (Systematic Review Center for Laboratory Animal Experimentation) Risk of Bias (RoB) tool for animal intervention studies (SYRCLE RoB) (Hooijmans et al., 2014). This approach assesses 10 bias domains adapted from the Cochrane RoB tool, including sequence generation, baseline characteristics, allocation concealment, randomization, performance bias, randomization of outcome assessment, blinding of outcome assessment, incomplete outcome data, reporting bias, and other sources of bias. Each domain of each study was judged as “Low,” “Unclear,” or “High” risk of bias. Any disagreements between reviewers were resolved through discussion until a consensus was reached. Cohen’s Kappa coefficient was used to measure the inter-rater reliability between the two reviewers to assess the consistency of judgments across the 10 domains of the SYRCLE Risk of Bias tool.

### Statistical analysis

Due to the heterogeneity of the outcome measures, a meta-analysis approach was not used in this study. Rather, a descriptive synthesis was used to summarize the findings.

## Results

### The search process

**Figure 1:**
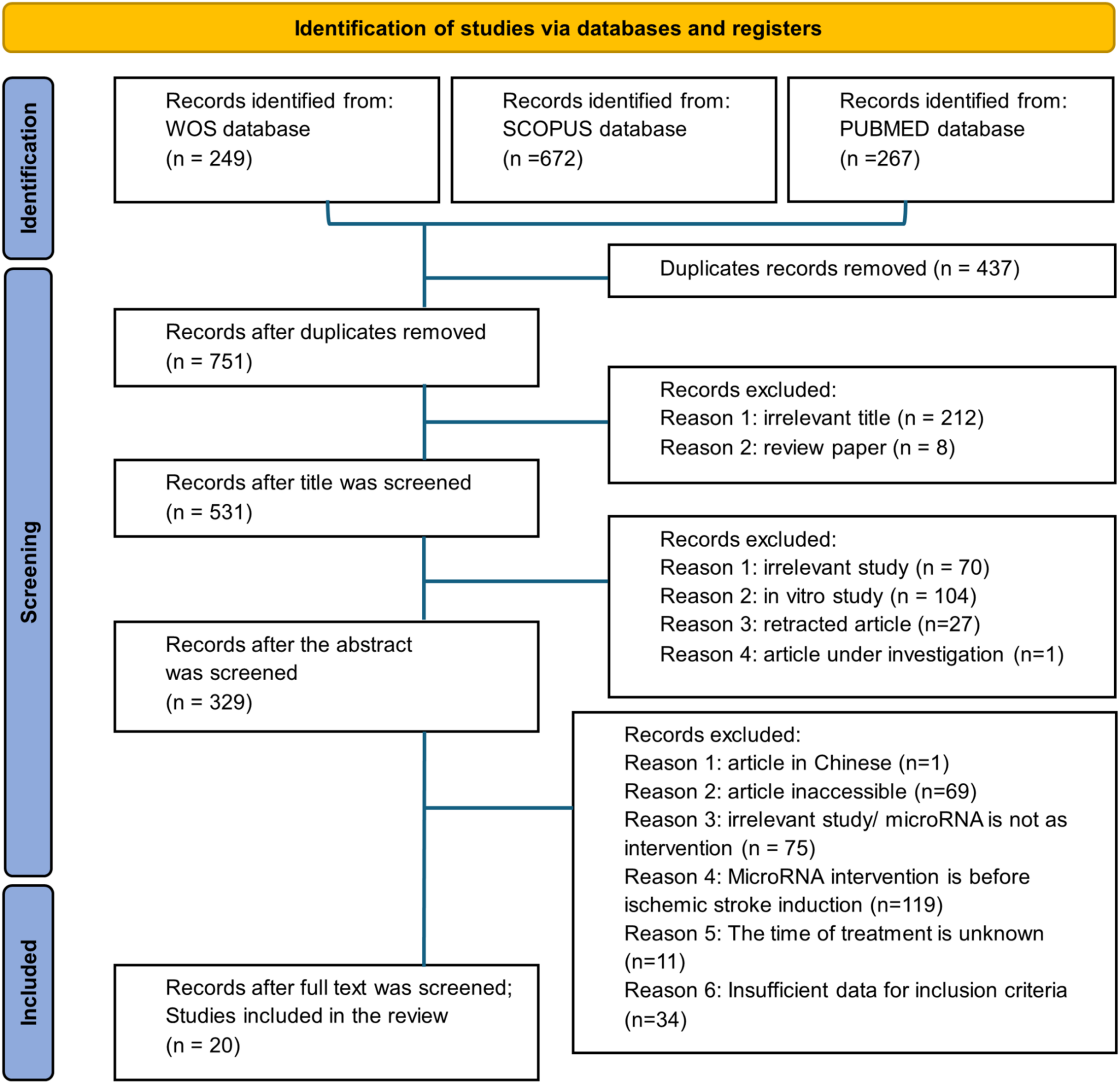
Flowchart of the search process using the PRISMA method.

The implemented search strategy identified 1,188 titles and abstracts from three databases. After filtering for article/full-text and English-only, 249 articles from Web of Science (WoS), 672 from Scopus, and 267 from PubMed were retrieved. A total of 437 duplicates were removed, and the remaining 751 records proceeded for title screening. A total of 220 articles were excluded at the title screening stage due to irrelevant titles (*n* = 212) and review studies (*n* = 8). Another 202 records were omitted after abstract screening due to irrelevant studies (*n* = 70), *in vitro* study design (*n* = 104), retracted articles (*n* = 27), and articles under investigation (*n* = 1). During the full-text screening, 69 records were excluded due to inaccessibility and one non-English article. Out of the full-text articles assessed for eligibility, 75 irrelevant studies were excluded. Pretreatment studies and studies involving genetically modified knockout or knockdown animals were not included in the current review (*n* = 119). Therefore, studies that did not provide the time for treatment injections were excluded (*n* = 11). Another 34 post-stroke miRNA treatment studies were excluded because the data provided were insufficient to meet the inclusion criteria. Finally, only 20 articles met the inclusion criteria and were selected for further analysis. Figure 1 shows the overall search process.

### Characteristics of the included studies

Based on 20 identified articles, studies of miRNA effects as post-treatment in animal models of ischemic stroke predominantly used rodents, either mice or rats. All studies except one have used middle cerebral artery occlusion, either transient or permanent, to stimulate cerebral infarction. The procedure commonly uses an intraluminal suture technique, which involves inserting a nylon- or silicone-coated suture under anesthesia. While few studies use pMCAO, reperfusion after 1 to 2 h of occlusion is more commonly used. One study induced stroke by double-ligation at 0.5 cm from the common carotid artery (CCA) bifurcation. Other methods of ischemic stroke induction, such as the drug-induced, photothrombotic, and embolism methods, were not cited in the selected studies. Euthanization of the animal for most studies is in the acute post-ischemia window (24–48 h), while the other minority euthanized animals at the subacute phase (2–14 days) or at the chronic phase (>14 days) of ischemic stroke.

Each study investigates the role of distinct miRNAs in ischemic stroke in rats by administering specific miRNA interventions. Several studies directly introduced synthetic miRNAs, such as agomirs, mimics, antagomirs, or inhibitors, while other experiments used exosomes enriched with miRNA intervention derived from various cell types. Some studies also administered other established agents, such as dexmedetomidine, coenzyme Q10, and pathway-specific inhibitors, to examine specific mechanisms. The route of administration is *via* intraperitoneal injection, intravenous injection *via* the tail, or intracerebroventricular injection using a microsyringe. The timing of intervention differs widely across studies; however, the drug was commonly administered immediately after tMCAO/pMCAO (0 to 2 h) or immediately after reperfusion. Some studies also utilized repeated injections on different days. Each miRNA manipulation produced distinct outcomes in cerebral infarcted animals, suggesting that these effects involve several independent mechanisms. The information in each study’s methodology was summarized according to Table 1.

**Table 1:** Characteristics of the selected articles in this study (*n* = 20).

Study no	Authors	Animal stroke model	MicroRNA intervention	Drug delivery & euthanization	Outcome and mechanism
1	Deng et al. (2020)	SD rats MCAO	miR-130a mimics miR-130a inhibitors XIAP interference plasmids	**r:** tail vein **t:** immediately after MCAO **e:** 24 h post-MCAO	Inhibition of miR-130a enhances neurological recovery, cognitive function and angiogenesis.
2	Pan et al. (2020)	C57BL/6 mice tMCAO 90 min	Mesenchymal stem cell (MSC)-derived exosomes miR-132-3p	**r:** tail vein **t:** 90 min post-tMCAO **e:** 48 h post-intervention	miR-1323p priming of MSC-derived exosomes protects against oxidative damage, apoptosis, BBB disruption, and brain infarction.
3	Wang et al. (2020b)	SD rats tMCAO 2 h	miR-22 LY294002	**r:** ICV **t:** 2 h post-tMCAO **e:** 24 h post-reperfusion	miR-22 overexpression reduces neurological deficits, brain infarction and induces angiogenesis.
4	Yi, Fang & Li (2020)	C57BL/6J mice tMCAO 1 h	miR-338-5p agomir	**r:** caudal vein **t:** after CIR injury **e:** 24 h post-reperfusion	miR-3385p reduces neurological deficits and infarct size *via* inhibiting apoptosis and inflammation.
5	Yue et al. (2020)	SD Rats tMCAO 2 h	miR-421 antagomir	**r:** ICV **t:** when reperfusion initiated **e:** 1 day post-reperfusion	Downregulation of miR-421 improves neurological function, reduces infarct size, suppresses neuronal apoptosis and oxidative damage.
6	Ghasemloo et al. (2021)	Wistar rats tMCAO 60 min	miRNA-149-5p mimic Q10	**r:** ICV **t:** 45 min post-tMCAO **e:** 24 h post-tMCAO	Upregulation of miR-1495p alone or in combination with CoQ10 offers neuroprotection against ischemic stroke and preserves the BBB.
7	Lv, Li & Che (2021)	C57BL/6 J mice tMCAO 1 h	Extracellular vesicles-miR-31 TRAF6	**r:** tail vein **t:** 1 h post-tMCAO **e:** 24 h post-reperfusion	miR-31-derived EVs improve neurological outcomes, reduce infarct size, and limit neuronal apoptosis.
8	Song et al. (2021)	C57/BL6 mice pMCAO	miR-140-5p agomir TLR4 plasmid	**r:** ICV **t:** 1 h post-pMCAO **e:** 48 h post-pMCAO	miR-140-5p reduces neurological deficits and infarct size while inhibiting neuronal apoptosis.
9	Song, Xue & Cai (2021)	C57BL/6 mice tMCAO 1 h	miR-181a-5p inhibitor	**r:** ICV **t:** post-tMCAO, before reperfusion **e:** 24 h post-reperfusion	miR-181a-5p downregulation or En2 overexpression reduces behavioral deficits, infarct size and apoptosis.
10	Wang et al. (2021)	SD rats tMCAO 2 h	miR-30a inhibitor	**r:** tail vein **t:** 90 min post-tMCAO **e:** 72 h post-reperfusion	miR-30a inhibition maintains BBB integrity, thus reducing infarct volume and improving neurological outcomess.
11	Xin et al. (2021)	C57BL/6 mice MCAO	Lentiviral-Anti-miR-23b Lentiviral-oe-c-Myc Lentiviral-miR-23b	**r:** ICV **t:** 1 h post-MCAO **e:** 24 h post-MCAO	Suppressing miR-23b reduces oxidative stress and apoptosis. The mice demonstrated smaller infarcts and enhanced neurological and cognitive recovery.
12	Yang et al. (2022)	C57BL/6J mice tMCAO 1 h	Extracellular vesicles-miR-155a-5p agomir Extracellular vesicles-miR-155a-5p antagomir Rheb	**r:** tail vein **t:** immediately after reperfusion, for 3 days **e:** 3 days post-tMCAO	miR-1555p inhibition or Rheb restoration reduces autophagy, inflammation, infarct volume, and improves neurological function.
13	Zhu et al. (2024)	C57BL/6J mice tMCAO 1 h	Exosomes-miR-486-5p mimics	**r:** tail vein **t:** 2 h post-reperfusion **e:** not reported	MSCs-derived exosomes enriched with miR-4865p, significantly improve neurological outcomes, reduce infarct size, and curb apoptosis.
14	Jin et al. (2023)	SD rats tMCAO 90 min	miR-199a-5p agomir miR-199a-5p antagomir	**r:** ICV **t:** 5 days post-tMCAO **e:** 14 days post-tMCAO	miR-199a-5p overexpression drives neurogenesis, reduces infarct size, and improves functional outcomes.
15	Zhu et al. (2021)	SD rats tMCAO 2 h	miR-199a antagomir Dexmedetomidine	**r:** ICV **t:** 0.5 h before reperfusion **e:** 24 h post-reperfusion	DEX neuroprotective effects will be diminished if miR-199a is restored. DEX improves neurological performance and inhibits autophagy & apoptosis.
16	Zhang et al. (2020)	SD rats tMCAO 1 h	miR-199a-5p inhibitor IGF-1	**r:** ICV **t:** 30 min post-tMCAO, once every 3 days **e:** 7 days post-tMCAO	miR-199a-5p inhibition improves cognition and neurological scores, reduces infarct size and decreases hippocampal apoptosis.
17	Lijuan et al. (2023)	c57 mice double-ligation at 0.5 cm from the CCA bifurcation	miR-210 antagomir miR-210 agomir 2-methoxyestradiol (2ME2)	**r:** intraperitoneal **t:** 24 h post-stroke **e:** 2 weeks post-stroke	Overexpression of miR-210 suppresses inflammation, reduces infarct size and improves neurological outcomes.
18	Wang et al. (2024)	C57BL/6 J mice pMCAO	miR-210-endothelial progenitor cell exosomes ly294002 su1498 k252a	**r:** caudal vein **t:** 2 h post-pMCAO **e:** 2 days post-pMCAO	Upregulation of miR-210 in ischemic brain tissue reduces infarct volume, improves neurological deficits and decreases neuronal apoptosis.
19	Wang et al. (2020a)	db/db type II diabetic mice MCAO	Endothelial progenitor cells-exosomes miR126	**r:** tail vein **t:** 2 h post-MCAO **e:** day 2 & day 14 post-intervention	miR-126-enriched EPC exosomes improve neurological function, reduce apoptosis and promote angiogenesis.
20	Xu et al. (2023)	C57BL/6 J mice pMCAO	Mixed infusion of neural progenitor cells-exosomes s miR-210 + endothelial progenitor cell-exosomes miR-126 k-252a	**r:** tail vein **t:** 2 h post-pMCAO **e:** 24 h post-intervention	Enhanced exosome therapy, combining NPC-derived miR-210 and EPC-derived miR-126 reduces infarct volume, improves neurological scores, decreases apoptosis, and reduces reactive oxygen species (ROS).

**Note:**

SD rats, Sprague Dawley rats; r, route of drug delivery; t, treatment time; e, euthanization time; ICV, intracerebroventricular injection.

### Expression of miRNAs after ischemic stroke in *in vivo* models

The expression of specific miRNA in *in vivo* models, as summarized in Table 1, demonstrates that several miRNAs are differentially regulated following ischemic injury. After an ischemic stroke attack, some miRNAs are demonstrated to be upregulated, and some miRNAs are downregulated in the blood serum or brain tissue, as proved *via* RT-PCR. Among the included studies, miR-199a-5p was upregulated in three studies, whereas two reported downregulation of miR-210. However, the included studies did not measure miR-132-3p, miR-30a, and miR-126 levels in sham animals to compare with those in ischemic stroke animals. MiRNA intervention, such as an agomir or mimic, increases the expression of the specific miRNA, while an antagomir or inhibitor reduces its expression.

### The effects of miRNA modulation on neurological function and brain infarct volume of ischemic stroke animal models

MiRNA modulation consistently demonstrated significant influence on neurological outcomes and brain infarct size. Common neurological tests to assess neurological deficits after stroke include the modified neurologic severity score (mNSS), the Longa score, and sensorimotor tests (beam balance test, foot fault test). The volume of brain infarction was often measured by 2,3,5-triphenyltetrazolium chloride (TTC) staining. Neurological deficits correlate significantly with the volume of infarcted areas. Some miRNAs exert protective effects, whereas others contribute to increased tissue damage. Upregulation of certain miRNAs and downregulation of others resulted in reduced neurological deficits and brain infarct volumes after stroke. Conversely, overexpression of certain miRNAs or repression of others led to negative outcomes. A combination of more than one miRNA, such as miR-126 + miR-210, was also demonstrated to yield stronger protective effects. Combining specific miRNA modulation with other interventions, such as IGF-1 and dexmedetomidine, produces better outcomes. These results show that miRNA modulation directly affects ischemic stroke outcome in animals, and that these effects are mediated by specific mechanisms discussed in the following section.

Overall, the findings suggest that miRNA modulation exerts bidirectional effects on neurological outcomes, depending on the targeted miRNA. Upregulation of certain miRNAs was associated with improved neurological scores and reduced infarct volume, whereas others were linked to worsened neurological deficits and increased brain injury. This variation underscores the importance of considering each miRNA’s functional role when interpreting intervention outcomes. A more detailed functional classification and mechanistic interpretation are discussed in the later sections.

### The mechanisms of miRNAs after ischemic stroke

The pathological process of ischemic stroke alters miRNA expression, which eventually influences brain injury and recovery trajectories. The studies investigate the effects of miRNA modulation after ischemic stroke and hypothesize the mechanisms involved by measuring biological markers *via* various methods, including reverse transcription polymerase chain reaction (RT-PCR), Western blot, enzyme-linked immunosorbent assay (ELISA), terminal deoxynucleotidyl transferase dUTP nick end labeling (TUNEL) assay, immunofluorescence staining, and luciferase reporter assay. Mechanistically, each miRNA studied has different targets, and these modulations are linked to reduced oxidative stress, decreased inflammation, inhibited apoptosis, enhanced neurogenesis, increased angiogenesis, and maintained BBB integrity. Several researchers have elucidated the pathways involved in miRNA regulation post-ischemic stroke attack. Further details on the mechanism of each miRNA and its target gene or target protein are listed in Table 2.

**Table 2:** Mechanisms and effects of miRNAs post-stroke *in vivo*.

Study no	Authors	miRNA studied	miRNA level without inter-vention	miRNA level with inter-vention	Neuro-logical function	Infarct size	Target	Mechanism	Outcome
1	Deng et al. (2020)	miR-130a	↑	↓	↑	↓	↑ XIAP	↓caspase-3, Bax; ↑ Bcl-2 ↑NSC proliferation, neurotrophic factors and angiogenic factors	Reduce neuronal apoptosis and enhance angiogenesis
				↑	↓	↑			
2	Pan et al. (2020)	miR-132-3p	–	↑	↑	↓	↓ RASA1	Activate Ras/PI3K/Akt/eNOS pathway	Reduce ROS production, apoptosis and BBB dysfunction
3	Wang et al. (2020b)	miR-22	↓	↑	↑	↓	-	Activate PI3K/Akt signaling pathway ↑ CD34+ and VEGF+ microvessels ↑ VEGF and Ang-1	Improve neuroprotective & angiogenic functions
				↑ and LY294002	↓	↑			
4	Yi, Fang & Li (2020)	miR-338-5p	↓	↑	↑	↓	↓ CTGF	Activate AMPK/mTOR pathway	Suppress apoptosis & inflammatory response
5	Yue et al. (2020)	miR-421	↑	↓	↑	↓	↑ MCL1	↑ Mcl-1, Bcl-2; ↓ Bax ↑ SOD, ↓ MDA	Suppress cell apoptosis and oxidative stress
6	Ghasemloo et al. (2021)	miRNA-149-5p	↓	↑	↑	↓	–	Suppress inflammatory cytokine ((IL-6, TNF-α) and MMP2/9	Preserves BBB and decreases edema
7	Lv, Li & Che (2021)	miR-31	↓	↑	↑	↓	↓ TRAF6	↓ IRF5 ↓ cleaved caspase-3, Bax	Reduce neuronal cell apoptosis
8	Song et al. (2021)	miR-140-5p	↓	↑	↑	↓	↓ TLR4	↓NF-κB (TLR4/NF-κB axis) ↓ cleaved caspase-3, Bax; ↑Bcl-2	Inhibit neuronal apoptosis
9	Song, Xue & Cai (2021)	miR-181a-5p	↑	↓	↑	↓	↑ En2	Activate the Wnt/b-catenin pathway	Reduce apoptosis
10	Wang et al. (2021)	MiR-30a	–	↓	↑	↓	↑ ZnT4	Activate ZnT4/zinc signaling pathway ↓ intracellular zinc preserves tight junctions	maintain BBB integrity
11	Xin et al. (2021)	miR-23b	↑	↓	↑↑	↓↓	↑ Nrf2	c-Myc negatively regulated miR-23b expression ↑Bcl2; ↓Bax, cleaved caspase3	Reduce neuronal apoptosis
				↑ and c-Myc	↑	↓			
12	Yang et al. (2022)	miR-155a-5p	↑	↑	↓	↑	Rheb	↑ miR-155-5p, ↓ Rheb, ↓ mTORC1 pathway, ↑ autophagy and ↑ NLRP3 inflammasome ↑ LC3-II/I, Beclin-1, LAMP-1, IL1β, IL18 Rheb/mTOR/NLRP3 pathway	miR-155a-5p induce autophagy and inflammasome
				↓	↑	↓			
13	Zhu et al. (2024)	miR-486-5p	↓	↑	↑	↓	↓ PTEN	↓ LDH, MDA; ↑ SOD activity	Inhibit apoptosis
14	Jin et al. (2023)	miR-199a-5p	↑	↑	↑	↓	↓ Cav-1	↑ VEGF and BDNF ↑ BrdU+/DCX+ and BrdU+/NeuN+ cell counts	Enhance neurogenesis
				↓	↓	↑			
15	Zhu et al. (2021)	miR-199a	↑	↓	↑	↓	-	DEX inhibit miR199a expression ↓ Beclin1, LC3B, cleaved caspase3; ↑p62 ↑ LC3B/NeuN co-staining	DEX inhibited autophagy and apoptosis
				↓ and DEX	↑↑	↓↓			
16	Zhang et al. (2020)	miR-199a-5p	↑	↓	↑	↓	↑ AKT	Activates AKT/mTOR signaling ↓Bax; ↑Bcl-2	Decrease neuronal apoptosis
				↓ and IGF-1	↑↑	↓↓			
17	Lijuan et al. (2023)	miR-210	↓	↑	↑	↓	HIF-1α	Inhibit HIF-1α/iNOS pathway ↓ IL-6 and TNF-α	Reduce inflammatory reaction
				↓	↓	↑			
18	Wang et al. (2024)	miR-210	-	↑	↑↑	↓↓	-	Activate VEGFR2/PI3K and TrkB/PI3K signaling cascades The effects are PI3K-dependent, with additional modulation by VEGFR2 and TrkB receptors	Reduce cellular apoptosis and oxidative stress
				↑ and ly294002	↓↓	↑↑			
				↑ and su1498	↑	↓			
				↑ and k252a	↑	↓			
19	Wang et al. (2020a)	miR126	–	↑	↑	↓	-	↓ cleaved caspase-3 ↑ VEGFR2 ↑ CBF and MVD	Reduce apoptosis and promote angiogenesis & neurogenesis
20	Xu et al. (2023)	miR-210 and miR-126 (combo)	↓ (both miRNA)	↑ (both miRNA)	↑	↓	–	↓ Nox2 ↑ BDNF/TrkB signaling	Reduce neural apoptosis, ROS production, and enhance dendritic integrity

### Risk of bias (RoB) assessment

**Figure 2:**
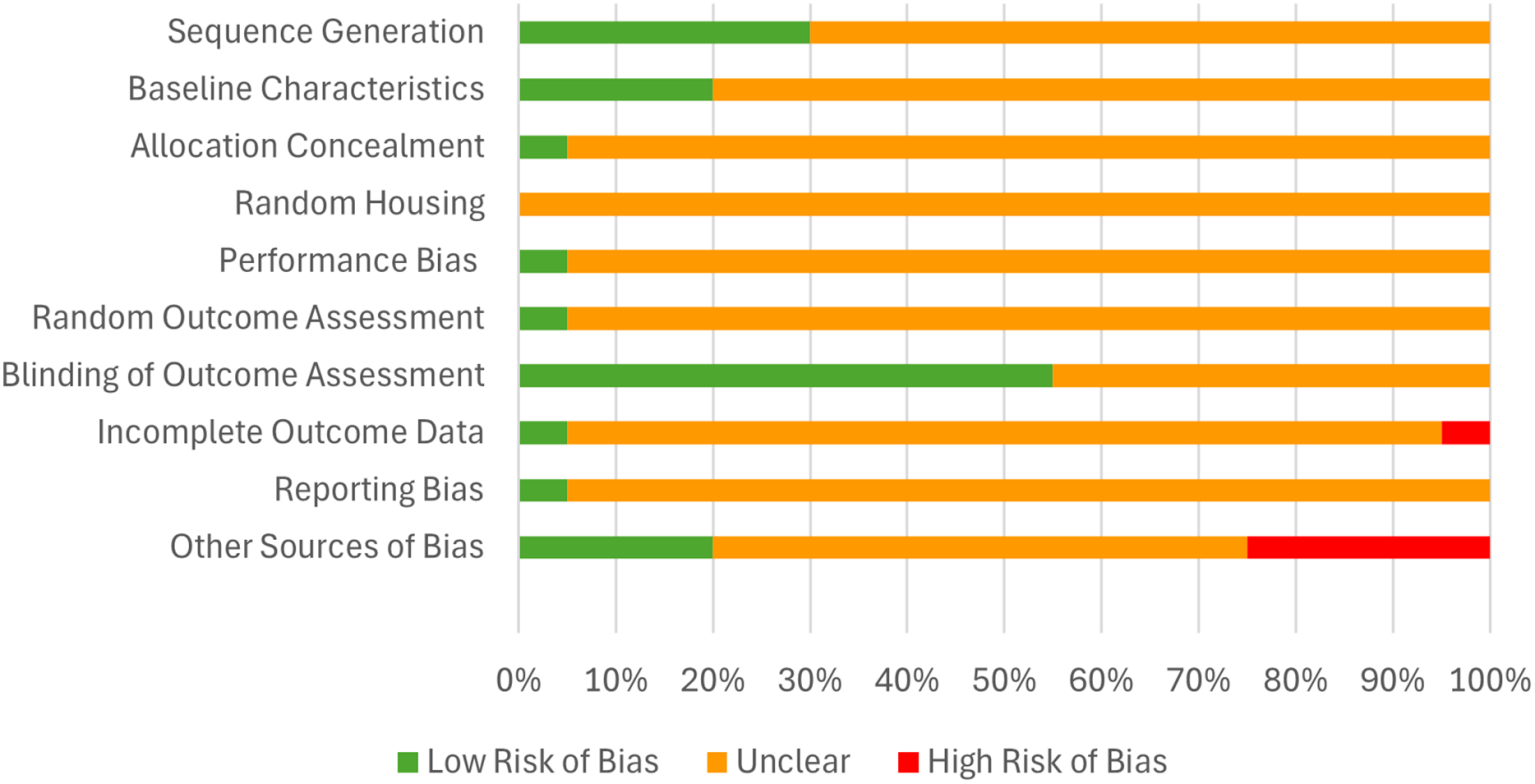
SYRCLE RoB assessment across all included studies.

All included studies were analyzed using the SYRCLE tool, and the result is summarized in Fig. 2. Overall, the risk of bias across the included studies was heterogeneous and predominantly characterized by unclear risk due to incomplete reporting. Most preclinical studies lacked sufficient methodological information to be confidently assessed as low risk across multiple domains. Only one study was categorized as low risk of bias, while the majority were rated unclear across several domains.

More than 50% of studies reported blinded assessments, while around 30% had a low risk of bias in sequence generation. However, the remaining domains were largely classified as unclear. Importantly, a proportion of studies demonstrated a high risk of bias in specific domains, particularly in incomplete outcome data and other sources of bias. About 25% of studies were rated as high risk under other sources of bias, mainly due to methodological concerns such as small sample sizes, unclear sample reporting, and potential methodological limitations. These findings highlight that the overall quality of evidence is limited not only by potential bias but also by insufficient reporting, which may affect confidence in the robustness of the findings. The detailed SYRCLE RoB ratings for each individual study are provided in Table S1.

The inter-rater reliability between the two independent reviewers was evaluated using Cohen’s Kappa based on the SYRCLE Risk of Bias tool. The observed agreement (Po) was 0.96 (96%), while the expected agreement (Pe) was 0.70 (70.1%). The unweighted Cohen’s Kappa was 0.87, and the weighted Cohen’s Kappa was 0.92, indicating almost perfect agreement between the two reviewers according to the Landis & Koch (1977) interpretation scale. Detailed results of the inter-rater reliability analysis are presented in Table S2.

## Discussion

The following discussion primarily synthesizes findings derived from the final 20 included studies summarized in Table 1, while integrating selected background literature to support mechanistic interpretation.

### Overview of miRNAs in ischemic stroke pathophysiology in animal models

A blockage of blood flow to the brain initiates a complex pathological cascade, including oxidative stress, apoptosis, inflammation, and tissue remodeling. MiRNAs are significant molecular regulators of these processes. Each miRNA can interact with multiple messenger RNAs, thereby influencing entire signaling networks. Studies using animal models have demonstrated dynamic alterations in miRNA expression and their regulatory roles in the onset, progression, and recovery of ischemic stroke.

During stroke onset, miRNAs can affect cerebrovascular health and ischemic vulnerability by modulating endothelial cell function, the inflammatory response, and cell survival. For instance, downregulation of miR-126 in mouse models leads to endothelial dysfunction, hindered vascular repair, and increased susceptibility to ischemia (Wang et al., 2008). Likewise, some miRNAs are indirectly associated with stroke predisposition, such as miR-21, miR-155, and miR-210, by modulating vascular inflammation and atherosclerotic processes (Rink & Khanna, 2011).

The progression of brain damage after ischemic stroke was significantly influenced by miRNAs. MiRNAs regulate cell death, inflammatory responses, and the integrity of the blood–brain barrier (BBB). Specific miRNAs were found to be upregulated and downregulated in the rodent brain tissue after hours following cerebral ischemia (Zhao et al., 2021). For example, upregulated miR-21 inhibits pro-apoptotic genes, while upregulated miR-210 reduces oxidative injury and promotes endothelial cell viability (Lopez, Dempsey & Vemuganti, 2017; Wang et al., 2024). On the other hand, elevated miR-155 after stroke facilitates pro-inflammatory cytokine production, and elevated miR-497 levels lead to neuronal apoptosis (Yin et al., 2010; Zhang et al., 2020). The regulation of inflammation by miRNAs could affect the degree of tissue damage. Suppression of miRNAs that enhance inflammatory signaling, like miR-155 in rodents, decreases infarct size, while inhibition of anti-inflammatory miRNAs, such as miR-124, extends tissue damage (Liu et al., 2013b; Pena-Philippides et al., 2016). In addition, stroke progression could also be restricted by protecting the BBB. Upregulation of miR-34a in mouse tMCAO models resulted in BBB disruption, while restoration of miR-132-3p prevents barrier damage and diminishes edema (Hu et al., 2020; Pan et al., 2020).

Following ischemia, miRNAs play a vital role in facilitating neural repair, including angiogenesis, neurogenesis, and synaptic remodeling. During the recovery phase, pro-regenerative miRNAs such as miR-126 improve microvascular density and promote collateral circulation in mouse Middle Cerebral Artery Occlusion (MCAO) models (Wang et al., 2020a). MiR-124 supports the differentiation of progenitor cells into fully developed neurons and enhances integration into existing networks (Yang et al., 2017). In addition, miRNA modulation helps reduce glial scar formation and promotes axonal regeneration (Li et al., 2021; Xin et al., 2013).

### Temporal dysregulation of miRNAs post-stroke

The abrupt deprivation of oxygen and glucose in the brain triggers a series of pathological processes and significant changes in the brain’s miRNA profile. Altered miRNA expressions actively affect cell survival, inflammation response, the brain’s vasculature, and neural networks. Experimental studies have highlighted the recurring contrasting response of protective and harmful miRNAs after ischemic stroke was induced.

The timing of this miRNA dysregulation is closely linked to the pathophysiology of cerebral ischemia (Neag et al., 2022). The pathophysiological phases of cerebral ischemia in animal models remain unchanged compared to human patients; however, the temporal evolution of injury and repair is compressed in rodent (Fluri, Schuhmann & Kleinschnitz, 2015; Sicard & Fisher, 2009). The hyperacute phase of ischemic stroke in rodents started from the onset to roughly 6 h and was followed by the acute phase that lasted until 24 to 48 h post-stroke (Branyan et al., 2022; Habotta et al., 2025; Jurcau & Simion, 2022). MiRNA measurements within the first few hours after ischemia onset were not reported in any of the selected studies. During the acute phase, there is a general trend of upregulation of detrimental miRNAs and downregulation of beneficial neuroprotective miRNAs.

The acute phase is characterized by disruption of the BBB, reactive oxygen species production, and evolving apoptosis in peri-infarct regions (Okada et al., 2020). Some miRNAs are strongly activated by ischemic stress and contribute to the intensification of pathological processes. For instance, miR-155-5p, miR-421, and miR-130a are upregulated in ischemic tissue, stimulate the NLRP3 inflammasome, amplify oxidative damage, and undermine cell survival, respectively (Deng et al., 2020; Yang et al., 2022; Yue et al., 2020). MiR-181a-5p and MiR-23b both increase and enhance apoptosis (Song, Xue & Cai, 2021; Xin et al., 2021).

In contrast, studies showed reduced expressions of a cluster of beneficial miRNAs that support endothelial stability, inhibit inflammation, and encourage neuronal survival. For example, downregulation of miR-210, a regulator that restricts oxidative damage, and miR-338-5p, which normally constrains inflammation (Xu et al., 2023; Yi, Fang & Li, 2020). Similarly, miR-140-5p and miR-31, which attenuate pro-apoptotic signaling, are both downregulated, thus exposing the brain to greater injury (Lv, Li & Che, 2021; Song, Xue & Cai, 2021). MiRNAs that promote angiogenesis, such as miR-22 and miR-126, both decline after ischemia, reducing the brain’s perfusion recovery (Wang et al., 2020a, 2020b; Xu et al., 2023).

The pro-apoptotic miRNAs remain elevated while miRNAs that support cellular survivability and vascular integrity stay suppressed. Together, these changes lead to neuronal loss, high microglial activation, and significant vascular impairment. In the subacute phase, from roughly 2 to 14 days post-stroke, delayed apoptosis and robust neuroinflammation continue, while the BBB repair mechanism is initiated, including angiogenesis (Jurcau & Simion, 2022; Shibahara et al., 2020). While limited data are available for beneficial miRNAs in this phase, studies have recorded increased expression of miR-155a-5p and miR-199a-5p, which encourage inflammation and apoptosis, at the 3^rd^ and 7^th^ days post-stroke, respectively (Yang et al., 2022; Zhang & Zhou, 2020).

Relatively few data in the present study address the chronic phase, defined as ≥14 days after stroke. The chronic phase is characterized by glial scar formation around the infarcted area, mild inflammation and oxidative stress, continued vascular remodeling, and neural network reorganization (Jurcau & Simion, 2022; Zbesko et al., 2018). During this phase, miR-199a-5p upregulation enhances neurogenesis while pro-inflammatory miR-210 expression is reduced (Jin et al., 2023; Lijuan et al., 2023).

**Figure 3:**
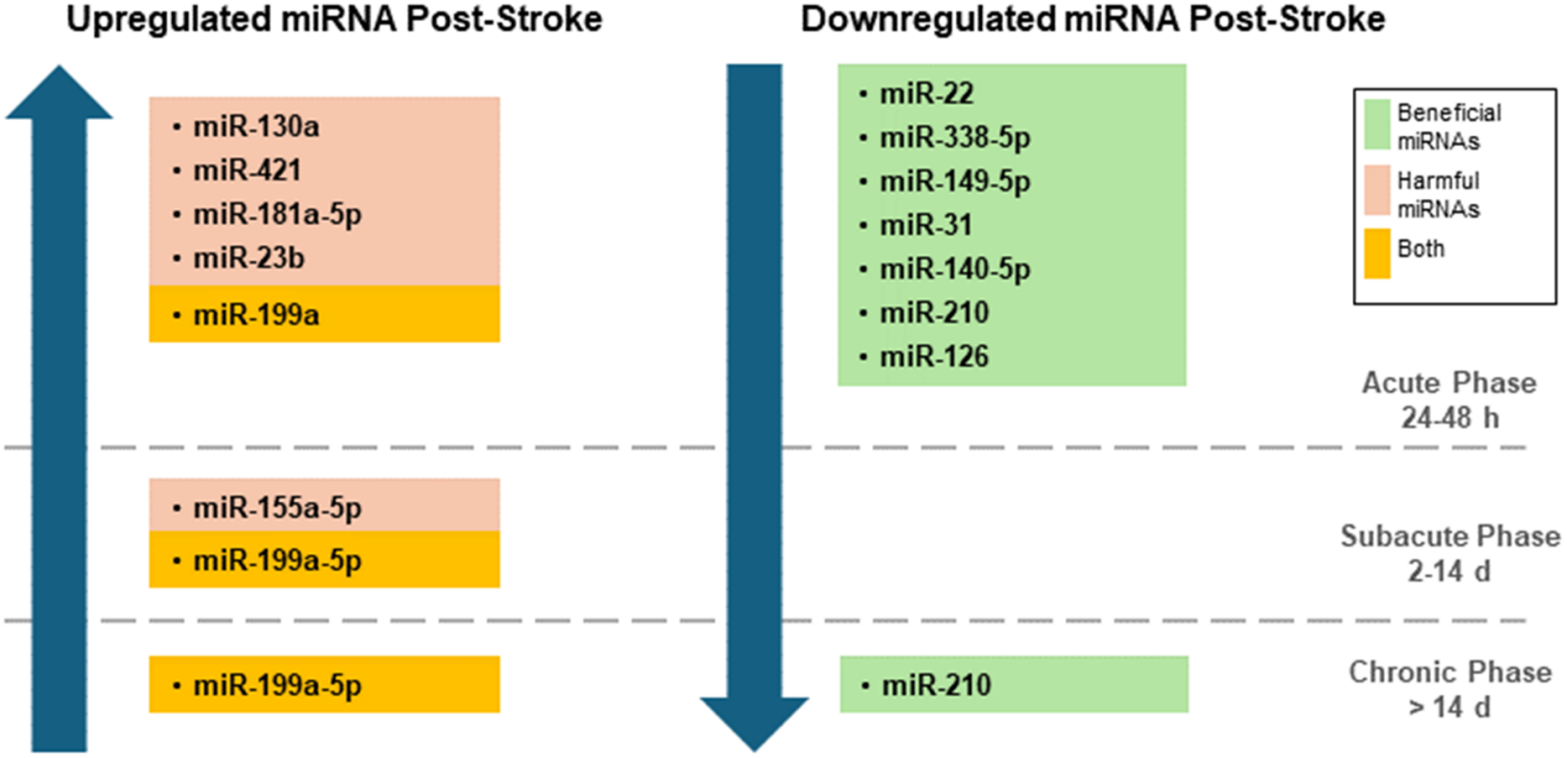
Temporal dysregulation of miRNA expression after ischemic stroke *in vivo*.

Figure 3 illustrates the upregulated and downregulated miRNAs across the acute, subacute, and chronic phases of ischemic stroke, and they were categorized as beneficial, harmful, or both. One study reported increased expressions of miR-486-5p post-stroke; however, incomplete data prevent its categorization into a specific post-ischemic phase (Zhu et al., 2024). The available evidence suggests that the optimal period for miRNA-based intervention is not a single point but rather a continuum. Therapeutic strategies in the early stage of ischemia may aim to antagonize detrimental miRNAs and, concurrently, restore protective miRNAs. Interestingly, members of the miR-199a family are upregulated across all three phases and exert dual, contrasting effects, thereby posing a therapeutic challenge. While miR-199a is observed in the acute phase, its mature strand, miR-199a-5p, predominates in later phases, suggesting context-dependent regulation across the temporal progression of injury and recovery. MiR-199a-5p is pro-apoptotic in the acute phase, yet promotes neurogenesis in the chronic phase (Lijuan et al., 2023; Zhang & Zhou, 2020; Zhu et al., 2021). Additionally, miRNAs that persistently down-regulate or up-regulate, such as miR-210, may serve as therapeutic targets throughout the phases since their normalization could mitigate ongoing oxidative stress and enhance long-term vascular plasticity. These findings highlight that miRNA function cannot be interpreted in isolation, but must be considered within specific temporal phases, cellular environments, and signaling contexts.

### Beneficial miRNAs: mechanism of neuroprotective effects post-stroke

A group of miRNAs can act as neuroprotective agents by lessening brain tissue damage and enhancing recovery after cerebral ischemia. These miRNAs regulate multiple interconnected pathways and influence various cell populations within the neurovascular unit, providing immediate protection and sustained repair.

#### Anti-apoptotic

One of the main ways miRNAs provide neuroprotection is by acting as key post-transcriptional regulators of apoptosis. Both intrinsic and extrinsic pathways of apoptosis are triggered following cerebral ischemia and reperfusion (García-Pupo et al., 2020). MiRNAs regulate the networks that determine mitochondrial integrity, caspase activation, and Bcl-2 family proteins. For example, miR-338-5p downregulates the target connective tissue growth factor (CTGF), thereby modulating the AMPK/mTOR axis. This finding was supported by an experiment using the Neuro-2a cells hypoxia/reperfusion model. Overexpression of miR-338-5p caused lower Bax protein, increased Bcl-2 protein, and supported cell survival (Yi, Fang & Li, 2020). Additionally, MiR-132-3p exerts anti-apoptotic effects in neurons by targeting and reducing RASA1, thereby stimulating Ras/PI3K/Akt signaling and downstream activation of eNOS. Eventually, the apoptotic responses and Reactive oxygen species (ROS) generation decrease in miR-132-3p-treated tMCAO mice (Pan et al., 2020).

Moreover, anti-apoptotic miR-31 binds to the 3′UTR of TRAF6 and suppresses its expression, including the downstream IRF5 signaling. This leads to inhibited neuronal apoptosis in the cortex and hippocampal CA1 regions of tMCAO mice, as measured by TUNEL staining. The result was also supported by a reduced expression of Bax, total caspase-3, cleaved caspase-3, and increased expression of Bcl-2 in miR-31-treated OGD neurons (Lv, Li & Che, 2021). In addition, miR-140-5p agomir in pMCAO mice directly downregulates TLR4 expression and suppresses NF-κB signaling, thereby reducing neuronal apoptosis and infarct size. Similar results were observed in OGD-induced neurons, including decreased Bax, reduced cleaved caspase 3, and increased Bcl-2 levels (Song et al., 2021). On the other hand, miR-486-5p directly targets and inhibits PTEN expression, reduces apoptosis, lowers brain damage, and improves neurological function in tMCAO mice. The outcome was supported by low levels of apoptotic proteins and flow cytometry analysis in an *in vitro* model (Zhu et al., 2024).

MiR-126 also protects the neurovascular unit against ischemic apoptosis, as demonstrated by a reduced number of TUNEL-positive cells and downregulation of cleaved caspase-3 in peri-infarct tissue of both diabetic and non-diabetic ischemic stroke mice (Wang et al., 2020a). In addition, miR-210 provides neuroprotection by upregulating the VEGFR2/PI3K and TrkB/PI3K signaling cascades. The anti-apoptotic benefits are partially blocked by VEGFR2 and TrkB inhibitors. Fewer apoptotic cells were detected in the peri-infarct brain of mice treated with miR-210 compared to the control group (Wang et al., 2024). Interestingly, the combination of miR-210 and miR-126 in exosomes simultaneously stimulates the BDNF/TrkB pathway while inhibiting the Nox2/ROS pathway, thereby resulting in a synergistic anti-apoptotic effect in neurons after stroke. This outcome was demonstrated in both animal and cellular models (Xu et al., 2023).

Despite targeting different upstream regulators, multiple miRNAs converge on common downstream apoptotic pathways, particularly the PI3K/Akt signaling cascade and Bcl-2 family regulation. This convergence highlights a shared neuroprotective mechanism across studies, resulting in reduced neuronal apoptosis and improved functional outcomes following ischemic injury.

#### Antioxidant

Massive production of reactive oxygen species occurs after ischemic stroke, particularly during the sudden reintroduction of oxygen during reperfusion (Sun et al., 2018). Several miRNAs exert antioxidant effects by activating endogenous defense systems or by interfering with ROS-generating components. For example, miR-132-3p, miR-210, and the combination of miR-210 and miR-126 (Pan et al., 2020; Wang et al., 2024; Xu et al., 2023). These miRNAs, which, in addition to their anti-apoptotic effects, also reduce ROS production in the peri-infarct area of stroke mice. Both miR-210 and miR-126 have been reported to protect from oxidative stress injury by down-regulating Nox2, and their combination significantly produces a more profound effect.

Although these miRNAs act through distinct molecular targets, their effects converge to reduce ROS production and enhance endogenous antioxidant defenses. This suggests a consistent pattern of antioxidant regulation across multiple experimental models.

#### Anti-inflammatory

MiRNA can also provide neuroprotection by modulating inflammation, the critical driver of secondary brain injury following ischemia. Vascular occlusion initiates the activation of resident microglia and astrocytes, as well as the rapid recruitment of peripheral immune cells (Kawabori & Yenari, 2015). Eventually, pro-inflammatory cytokines such as tumor necrosis factor-alpha (TNF-α), interleukin-6 (IL-6), and interleukin-1β (IL-1β) are elevated. For instance, overexpression of miR-210 in stroke mice significantly attenuates the inflammatory response, improves neurological score, and reduces infarct volume. MiR-210 modulates the levels of hypoxia-inducible factor 1α (HIF-1α) and inducible nitric oxide synthase (iNOS), thereby reducing the levels of TNF-α and IL-6 (Lijuan et al., 2023). Furthermore, miR-338-5p is predicted to bind with the 3′UTR of CTGF to suppress inflammatory response. Overexpression of miR-338-5p in Neuro-2a cells also led to a significant reduction of inflammatory cytokines (Yi, Fang & Li, 2020).

Despite acting through distinct upstream mediators, these miRNAs collectively suppress key inflammatory cytokines, including TNF-α and IL-6. Collectively, these findings highlight a shared anti-inflammatory effect, suggesting that inflammation is a central mechanism underlying neuroprotection in ischemic stroke.

#### BBB-preserving

After a stroke attack, the blood-brain barrier (BBB) is severely compromised, leading to vasogenic edema, infiltration of peripheral immune cells, and secondary brain injury. This disruption is mainly caused by the increased activity of matrix metalloproteinases (MMPs) and pro-inflammatory cytokines, leading to the breakdown of the tight junction proteins (Candelario-Jalil, Dijkhuizen & Magnus, 2022). MiR-149-5p inhibits the inflammatory cascade and reduces the major trigger of BBB disruption by significantly decreasing MMP-2 and MMP-9 levels. The miR-149-5p-treated rat stroke model showed reduced Evans blue extravasation into the brain parenchyma and cerebral edema, indicating decreased BBB permeability (Ghasemloo et al., 2021). Likewise, miR-132-3p also reduced BBB dysfunction, as demonstrated by decreased extravasation of Evans blue dye, reduced brain water content, preserved cerebral microvascular density, and improved cerebral blood flow in the peri-infarct area of tMCAO mice (Pan et al., 2020).

Although different molecular targets are involved, these miRNAs consistently contribute to maintaining BBB integrity by suppressing matrix degradation and inflammatory signaling. This highlights a unified protective effect on vascular stability across studies.

#### Pro-angiogenic

Some miRNAs demonstrate potent pro-angiogenic effects through distinct molecular pathways. Angiogenesis aids in restoring blood flow in the peri-infarct area, thus delivering oxygen and nutrients that promote the survival of threatened neurons and enhance neurogenesis. In a diabetic mouse stroke model, miR-126 administration leads to a significant increase in angiogenesis, as evidenced by the increased number of newly generated endothelial cells (CD31+ BrdU+) in the ischemic penumbra. The mice also showed improvements in cerebral microvascular density and cerebral blood flow, as well as long-term recovery of neurological function (Wang et al., 2020a). In the rat tMCAO model treated with miR-22, PI3K/Akt signaling is activated, leading to downstream upregulation of key pro-angiogenic factors, including vascular endothelial growth factor (VEGF) and Angiopoietin-1 (Ang-1) in serum. VEGF promotes endothelial cell proliferation, while Ang-1 stimulates vessel maturation and stability. The microvessel density and VEGF level were significantly higher in the cortex of miR-22-treated rats (Wang et al., 2020b).

Despite activating distinct signaling pathways, these miRNAs converge on promoting endothelial proliferation, vascular remodeling, and improved cerebral perfusion. This coordinated effect underscores the importance of miRNA regulation in post-stroke vascular recovery.

#### Neurogenic

In addition to maintaining the existing tissue, neuroprotective miRNAs also facilitate neurogenesis and enhance synaptic plasticity. MiR-199a-5p promotes endogenous neuronal differentiation by binding to caveolin-1 at the 3′UTR and inhibiting its activity, thereby increasing levels of neurotrophic factors such as Brain-Derived Neurotrophic Factor (BDNF) and VEGF. The peri-infarct region of miR-199a-5p-treated tMCAO rats showed a significant increase in the number of new, immature neurons (BrdU+/DCX+ cells) and mature neurons (BrdU+/NeuN+ cells) (Jin et al., 2023). While miR-199a-5p directly promotes neural stem cell differentiation into neurons, miR-126 indirectly enhances neurogenesis by promoting angiogenesis, thereby providing a well-vascularized niche that supports new neuron survival. The study showed that mice treated with miR-126 had a higher number of newly formed neurons (BrdU+/NeuN+ cells) and astrocytes (BrdU+/GFAP+ cells) in the peri-infarct area 14 days after stroke (Wang et al., 2020a).

Although they act through distinct molecular targets, these miRNAs consistently enhance neuronal differentiation and integration. This suggests that miRNA-driven neurogenesis is a convergent mechanism supporting long-term functional recovery after ischemic stroke.

### Summary

In sum, neuroprotective miRNAs function as coordinated regulators across multiple stages of ischemic stroke, from acute injury to long-term recovery. While individual miRNAs act through distinct molecular targets, a key unifying feature is their convergence on shared biological processes, including apoptosis suppression, oxidative stress reduction, inflammation control, vascular stabilization, and tissue regeneration.

Importantly, these miRNAs do not act in isolation but collectively regulate interconnected signaling networks, particularly those involving PI3K/Akt, VEGFR2, and BDNF/TrkB pathways. This integrated regulation enables simultaneous modulation of neuronal survival, vascular repair, and neurogenesis.

The comparative analysis further highlights that miRNA-mediated neuroprotection represents a multi-target strategy, in which diverse upstream mechanisms ultimately converge to produce functional outcomes. This reinforces the concept that miRNAs serve as central coordinators of neurovascular recovery rather than isolated molecular effectors.

### Harmful miRNAs: mechanism of detrimental roles in stroke outcomes

While certain miRNAs provide neuroprotection by promoting repair and functional recovery, other miRNAs can produce harmful effects. These detrimental miRNAs can be quickly upregulated following ischemia and contribute to the pathology of stroke.

#### Pro-apoptotic

Many miRNAs have been identified as anti-apoptotic regulators; however, others exert pro-apoptotic effects and contribute to neuronal injury following ischemic stroke. For example, miR-130a induces apoptosis by targeting and suppressing the X-linked inhibitor of apoptosis protein (XIAP), which inhibits caspases 3, 7, and 9. Downregulation of XIAP increases caspase activity and programmed cell death in neurons and endothelial cells. Inhibition of miR-130a in the rat stroke model resulted in lower caspase-3 activity, decreased Bax protein, and increased Bcl-2 protein, indicating that neuronal apoptosis was suppressed. The rat also exhibits improved neurological function and reduced infarct size (Deng et al., 2020). In addition, miR-421 is another miRNA that is upregulated after ischemic stroke and is pro-apoptotic. MiR-421 targets and downregulates myeloid cell leukemia-1 (MCL1), an anti-apoptotic member of the Bcl-2 protein family. MiR-421 antagomir administered in stroke rats caused increased MCL1, elevated Bcl-2, and decreased Bax, indicating miR-421 facilitates the activation of Bax-dependent apoptotic cascades (Yue et al., 2020).

Moreover, miR-181a-5p shuts down the critical neuroprotective signaling pathway by inhibiting engrailed-2 (En2), thereby deactivating the Wnt/β-catenin pathway. This pathway is crucial for neuronal survival and neurogenesis during ischemia. Inhibition of miR-181a-5p in tMCAO mice significantly reduced the cerebral infarction volume, decreased apoptotic neurons, and improved neurological scores (Song, Xue & Cai, 2021). On the other hand, miR-23b suppresses the cellular antioxidant defense system. MiR-23b directly downregulates nuclear factor erythroid two-related factor 2 (Nrf2), which controls the expression of numerous antioxidant genes. However, miR-23b expression is negatively regulated by c-Myc. In a stroke mouse model, silencing miR-23b reduced neuronal apoptosis, as demonstrated by TUNEL staining, and decreased the level of apoptotic proteins (Xin et al., 2021). Additionally, miR-155-5p exacerbates ischemic brain injury by promoting cell death *via* autophagy. MiR-155-5p targets and suppresses Ras homolog enriched in brain (Rheb), and thereby deactivates the mTOR signaling pathway, a key regulator of cell proliferation and survival. Delivery of miR-155-5p in ischemic mice inhibits Rheb, increases autophagy, and reduces neuronal viability (Yang et al., 2022).

The previously mentioned role of miR-199a-5p in neurogenesis is in contrast to studies that demonstrated a pro-apoptotic role of miR-199a (Jin et al., 2023). A group of researchers showed that DEX treatment to cerebral infarcted rats inhibits miR-199a and improves neurocyte injury, thereby suggesting miR-199a promotes both autophagy and apoptosis. The study demonstrated miR-199a inhibition caused a significant reduction in expression levels of Beclin1, LC3B, and cleaved caspase-3, while an increase in p62 protein levels (Zhu et al., 2021). Similarly, another study demonstrated that miR-199a-5p directly targets AKT protein and eventually promotes apoptosis. Inhibition of this miRNA resulted in activated AKT/mTOR signaling pathway, increased neuronal survival, and reduced infarct area (Zhang & Zhou, 2020). This discrepancy suggests that miR-199a-5p has cell-type-specific, context-dependent functions. MiR-199a may promote apoptosis in mature neurons by inhibiting AKT, while simultaneously enhancing neural stem cell differentiation by inhibiting Cav-1.

Despite targeting distinct upstream regulators such as XIAP, MCL1, AKT, and Nrf2, these miRNAs converge to amplify apoptotic signaling pathways, particularly by activating caspases, altering Bax/Bcl-2 balance, and suppressing cell survival pathways. This convergence highlights a shared detrimental mechanism leading to increased neuronal cell death and worsened functional outcomes following ischemic stroke.

#### Pro-oxidant and pro-inflammatory

In addition, the detrimental role of miRNA following ischemic stroke exacerbates ROS-mediated damage. MiR-421 enhances oxidative stress by suppressing the expression and activity of the antioxidant enzyme superoxide dismutase (SOD), specifically SOD-2. Concurrently, the level of the oxidative stress marker, malondialdehyde, also increased. The effects were demonstrated in both an *in vitro* model using PC12 cells and an animal model using SD rats (Yue et al., 2020). Moreover, the miR-155-5p is not only involved in apoptosis but also enhances inflammation, further contributing to stroke pathology. By suppressing Rheb expression, the NLRP3 inflammasomes are excessively activated, leading to the production of pro-inflammatory cytokines such as IL-1β and IL-18. In mouse models, miR-155-5p agomir aggravated ischemic brain injury, intensified neuroinflammation, increased cerebral infarction rates, and worsened neurological deficits (Yang et al., 2022).

Although these miRNAs act through distinct molecular targets, their effects converge to enhance oxidative stress and inflammatory signaling, particularly by increasing ROS production and activating pro-inflammatory cytokines such as IL-1β and IL-18. This coordinated amplification of cellular stress responses contributes significantly to secondary brain injury following ischemic stroke.

#### BBB-compromising and anti-angiogenic

Harmful miRNAs, such as miR-30a, can also accelerate the breakdown of the blood–brain barrier following an ischemic event. MiR-30a directly binds to the 3′-UTR of zinc transporter protein (ZnT4) and decreases its expression. Eventually, the repression leads to an intracellular zinc overload in microvascular endothelial cells, thereby stimulating the degradation of tight junction proteins, such as occludin and claudin-5. Ultimately, the effects lead to increased BBB permeability, vasogenic edema, and the facilitation of inflammatory responses (Wang et al., 2021). In addition, the pro-apoptotic miR-130 impaired angiogenesis. MiR-130a mimics in stroke rats directly downregulate XIAP, causing a significant reduction in the number of new blood vessels in the brain tissue surrounding the lesion. The pro-angiogenic factors, such as VEGF and hepatocyte growth factor (HGF), are suppressed. Conversely, when miR-130a was inhibited, the number of new vessels increased as measured using CD31/α-SMA double staining. This indicates that lower levels of miR-130a promote angiogenesis (Deng et al., 2020).

Despite involving distinct molecular mechanisms, these miRNAs consistently disrupt vascular integrity and impair post-stroke repair processes. Their actions converge on increasing BBB permeability and inhibiting angiogenesis, thereby exacerbating edema, inflammation, and impaired tissue recovery following ischemic injury.

### Summary

In summary, detrimental miRNAs function as key drivers of ischemic injury by disrupting critical cellular homeostasis and repair mechanisms. Although individual miRNAs act through distinct molecular targets, a unifying feature is their convergence on shared pathological processes, including the activation of apoptosis, amplification of oxidative stress, inflammation, and vascular disruption.

These miRNAs collectively impair neuronal survival pathways, particularly by suppressing key regulators such as AKT, XIAP, MCL1, and Nrf2, while simultaneously activating inflammatory cascades, including the NLRP3 inflammasomes.

Importantly, these detrimental miRNAs do not act independently but interact across interconnected signaling networks, leading to compounded injury effects. This highlights that miRNA-mediated damage in ischemic stroke is not driven by isolated pathways but by coordinated dysregulation of multiple biological systems.

From a therapeutic perspective, targeting these miRNAs represents a multi-target strategy to restore cellular balance, reduce injury progression, and improve functional recovery following ischemic stroke.

**Figure 4:**
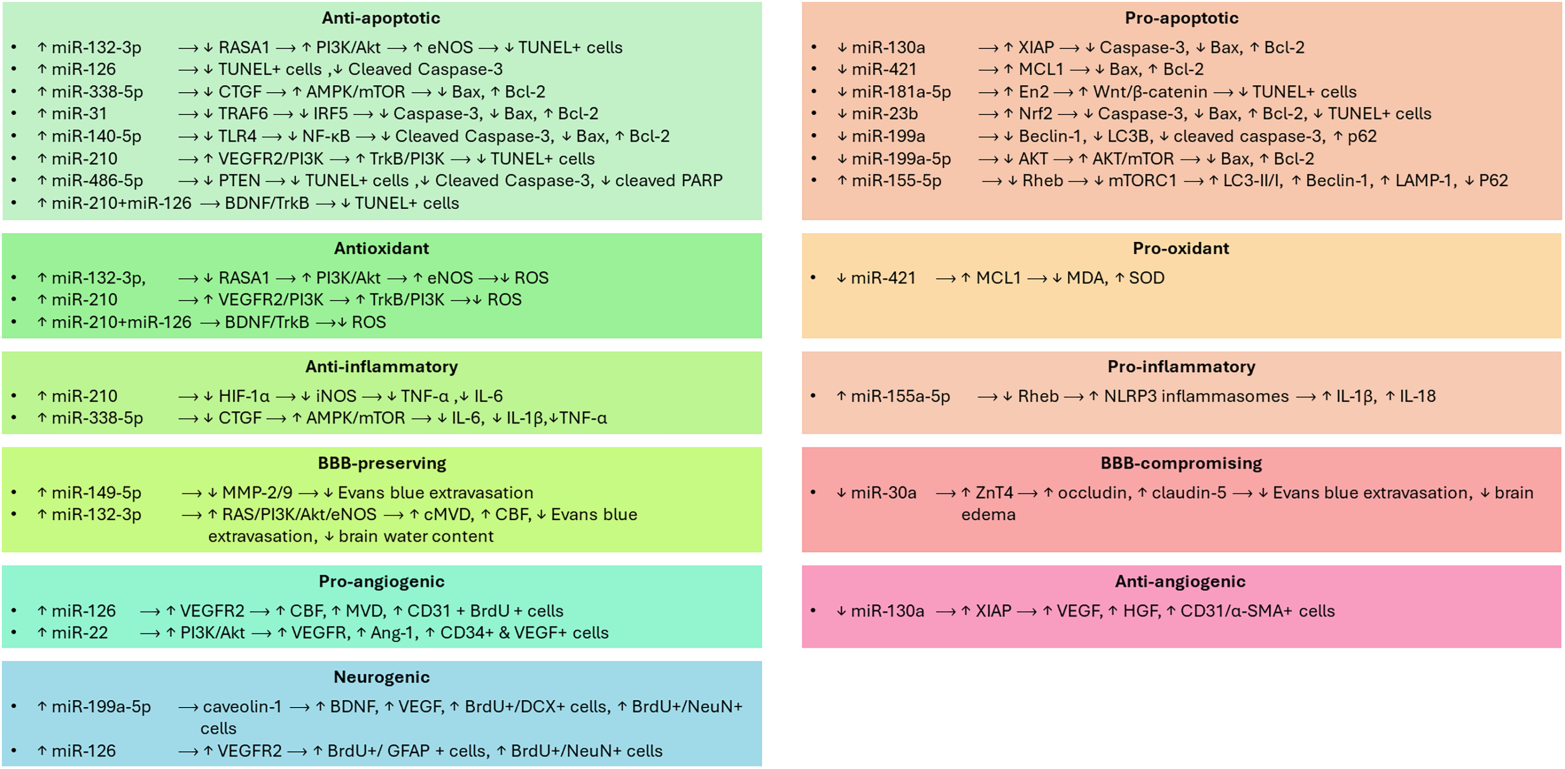
Mechanism of beneficial and harmful miRNA *in vivo* post-stroke.

**Figure 5:**
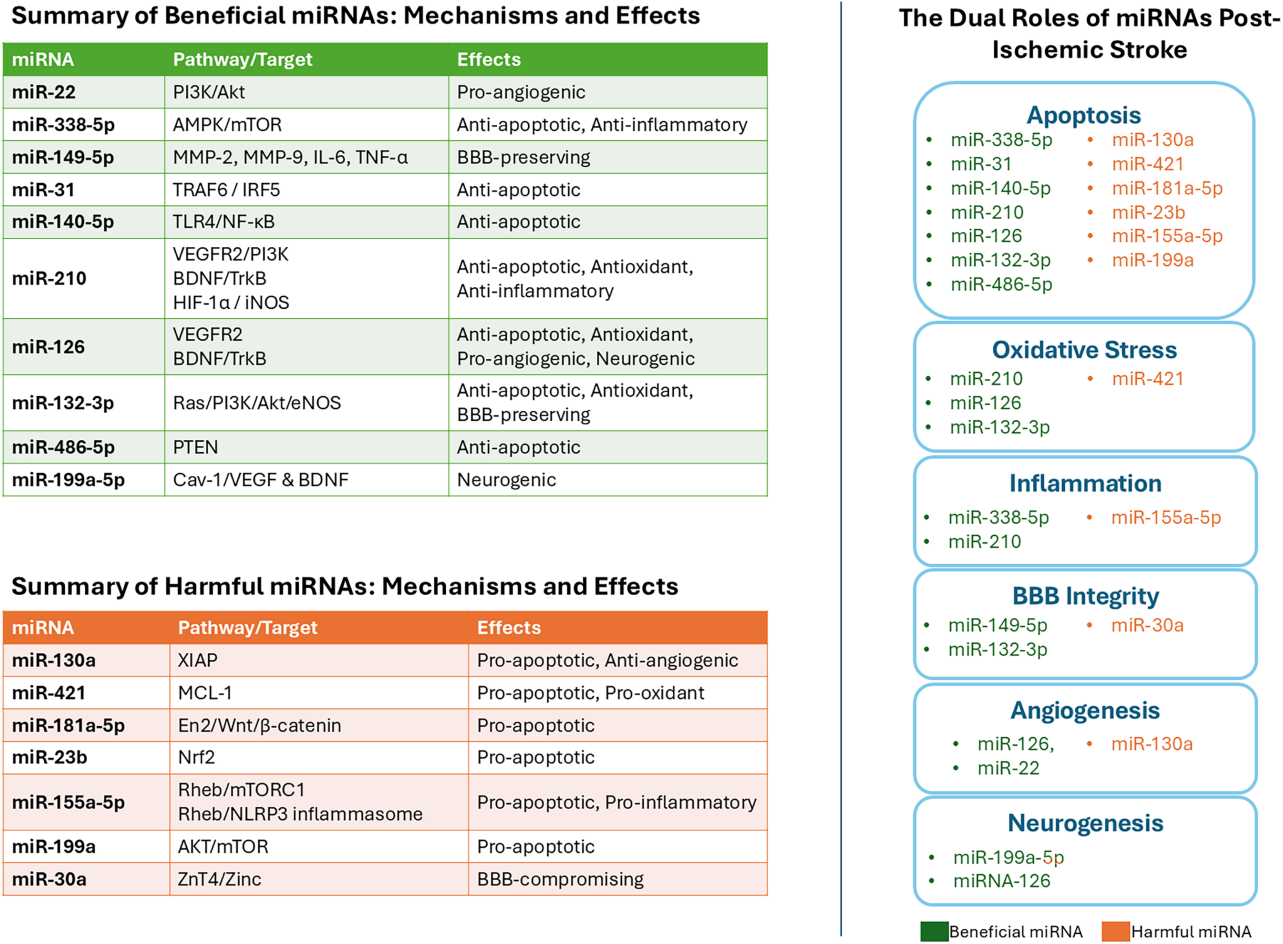
The mechanisms and effects of the dual roles of miRNAs post-stroke.

The mechanisms underlying both beneficial and detrimental miRNAs are summarized in Figs. 4 and 5. These figures highlight the convergence of multiple miRNAs on key biological processes, including apoptosis, oxidative stress, inflammation, BBB integrity, angiogenesis, and neurogenesis. Notably, both beneficial and harmful miRNAs regulate overlapping pathways, often in opposing directions. This reinforces the concept that ischemic stroke progression is governed by a dynamic balance between protective and detrimental miRNA-mediated mechanisms.

The biological processes involved in cellular injury, including apoptosis, oxidative stress, and inflammation, are tightly interconnected and co-regulated by multiple miRNAs. Rather than acting independently, these miRNAs function within integrated cellular signaling networks, in which a single miRNA can simultaneously influence multiple pathological pathways. In contrast, processes associated with tissue repair, such as angiogenesis, neurogenesis, BBB restoration, and others, are governed by more specialized yet highly coordinated miRNAs. This distinction highlights a functional asymmetry between injury amplification and repair mechanisms, further emphasizing the importance of restoring miRNA balance as a therapeutic strategy.

### MiRNA intervention approaches: synthetic modulators and exosome-based therapies

The studies have highlighted two primary strategies for miRNA-based intervention. MiRNAs can be administered as synthetic miRNA modulators or by using cell-derived exosomes as natural nanocarriers. Synthetic miRNA modulators are chemically synthesized oligonucleotides that are directly administered to animals to either mimic or inhibit the function of a target miRNA. Additionally, miRNA agomirs and antagomirs are optimized for stability and efficacy *in vivo* (Saenz-Pipaon & Dichek, 2023). For example, miR-149-5p mimic, miR-199a-5p agomir, and miR-181a-5p inhibitor were cited in these studies. The production of these synthetic miRNA modulators is straightforward and cost-effective; however, the administration to the animal usually requires direct intracerebroventricular injection (Tang & Khvorova, 2024).

Another way to intervene with miRNAs in animals is to administer exosomes as vehicles to deliver therapeutic miRNAs to target cells. This can be achieved by genetically modifying source cells to overexpress a specific miRNA, or by harnessing endogenous miRNAs from stem or progenitor cells. After the source cells were transfected with miRNA, their miRNA-enriched exosomes were harvested and administered to the animal. These biologically derived vesicles can be administered intravenously and possess a natural ability to cross the BBB, which will then be taken up by target cells such as neurons (Feng et al., 2025). Exosomes protect the miRNA from degradation and offer low immunogenicity; however, the production process is complex, costly, and difficult to standardize. In the current review, several studies have used this intervention approach, such as miR-132-3p mimics transfected into mesenchymal stromal cells and adipose-derived stem cells engineered to overexpress miR-31.

Researchers have also made efforts to combine the benefits of multiple cell types. For example, a method combining exosomes from neural progenitor cells rich in miR-210 with endothelial progenitor cells’ (EPC) exosomes containing miR-126 resulted in a synergistic effect. EPC exosomes containing miR-126 have been demonstrated to enhance VEGFR2 signaling, thus promoting cerebral angiogenesis and neurogenesis (Wang et al., 2020a) Another study involving miR-210–primed EPC exosomes resulted in reduced oxidative stress and cell death through the activation of both VEGFR2/PI3K and TrkB/PI3K signaling pathways (Wang et al., 2024). The combined miR-210 and miR-126 exosome therapy diminished ROS generation, reduced apoptosis, and increased dendritic spine density *via* the Nox2/ROS and BDNF/TrkB pathways. Remarkably, the effectiveness exceeds single-source exosome therapy in experimental stroke (Xu et al., 2023).

Based on data provided from selected studies, the cellular environment after stroke is regulated by a molecular conflict between two functionally opposite groups of miRNAs. The mechanism of both beneficial and harmful miRNAs is summarized in Fig. 4. Beneficial miRNAs employ multi-target mechanisms to mitigate inflammation, limit apoptosis, protect the BBB, and facilitate neurovascular remodeling. Conversely, detrimental miRNAs intensify brain injury by dismantling these same protective mechanisms, eventually amplifying the initial ischemic injury.

### Clinical implications and translational challenges

MiRNA treatments are shifting from single-target drugs to strategies that modulate whole biological systems. The abundance of preclinical data highlights the significant potential of targeted alteration of certain miRNAs. The potential of miRNA therapies depends on their ability to address the complex pathology of ischemic stroke holistically. Therapies that modulate miRNAs, such as miR-210 and miR-140-5p, have been shown to reduce infarct volume and prevent neuronal apoptosis (Song et al., 2021; Wang et al., 2024). In addition to acute neuroprotection, miRNA modulation may also support long-term repair processes essential for significant recovery. For instance, miRNAs including miR-22 and miR-126 have shown angiogenic capabilities in the ischemic penumbra to reestablish blood flow, while miR-199a-5p has demonstrated the ability to promote neurogenesis (Jin et al., 2023; Wang et al., 2020a, 2020b). Many of these miRNAs have been delivered *via* exosomes, natural nanocarriers that efficiently cross the BBB and deliver miRNA directly to damaged cells. Exosomes enriched in specific miRNAs offer an advanced, biologically suitable delivery system.

Despite this enormous potential, the issue of miRNA modulation in clinical translation is the specificity and targeting. The problems involve challenges in specifically delivering the treatment to the brain and in the precision of its molecular effect upon arrival. Preclinical studies typically use invasive injections; however, in clinical settings, the challenges posed by the BBB must be considered for effective systemic administration (Hanna, Hossain & Kocerha, 2019). Exosomes have demonstrated a natural capacity to address this issue; however, additional studies are required to enhance miRNA targeting to the ischemic penumbra and reduce unintended accumulation in other tissues (Diener, Keller & Meese, 2022). A single miRNA is capable of controlling hundreds of distinct messenger RNAs. This extensive therapeutic impact also poses a considerable risk of unintended consequences. For example, one study indicates that miR-199a-5p inhibition provides neuroprotection by blocking apoptosis through the AKT pathway, while another study shows that its upregulation aids neurogenesis by targeting Cav-1 (Jin et al., 2023; Zhang et al., 2020). This duality highlights the importance of understanding the functions of each miRNA, which are specific to cell types and context-dependent, to minimize detrimental off-target effects.

Additionally, there is a significant gap in the studies regarding the long-term effects and safety of these miRNA interventions. Most preclinical studies focus on short-term time points, typically 24 h to 14 days after stroke. Although the acute benefits are clear, the long-term effects are uncertain. MiRNA modulation may induce permanent changes in gene expression networks within the brain (Bartel, 2004; Conaco et al., 2006). Since stroke recovery is a prolonged process, long-term studies are important for verifying lasting functional improvements and for observing any delayed negative effects.

Moreover, the complex stroke pathology suggests that the therapeutic potential of miRNA modulation may be greater in rationally designed combination therapies. For example, combining a miR-199a-5p inhibitor with the AKT activator IGF-1 resulted in better outcomes than either treatment alone (Zhang & Zhou, 2020). Similarly, a treatment combining NPC-derived exosomes loaded with miR-210 with EPC-derived exosomes enriched in miR-126 provided improved neuroprotection (Xu et al., 2023). Therefore, future miRNA therapy might involve combining novel agents into comprehensive strategies that simultaneously target multiple injury and repair mechanisms. For example, combination therapies targeting multiple pathways, such as anti-inflammatory and pro-angiogenic miRNAs, may need to be identified.

### Study limitations

This review has several limitations. Firstly, the current study focuses only on peer-reviewed articles that examine the effects of miRNA modulation following ischemic stroke. Only studies that use animal models of ischemic stroke and were published in the last 5 years are involved. While numerous articles have been published on this topic over the past 15 years, this review could not analyze the entire field of miRNA research in ischemic stroke but focused only on the latest discoveries.

The review includes only preclinical studies essential for mechanistic discovery; however, the studies exhibit heterogeneity in their experimental designs. Researchers used varying durations of ischemia and reperfusion, different routes and timings of therapeutic administration, and a diverse set of behavioural and histological outcome measures. Therefore, the therapeutic efficacy and safety profiles may face challenges for clinical translation.

Furthermore, quantitative synthesis was constrained, as most included studies reported their findings, such as infarct volume and neurological scores, in graphical format without giving extractable numerical data. This limited the computation of standardized effect sizes and prevented a direct quantitative comparison of studies. Future studies should report numerical data to enable more robust quantitative synthesis. In addition, the ischemic model in some included studies is described as MCAO without specifying whether it was tMCAO or pMCAO, which can affect the interpretation of pathophysiological outcomes. Another limitation is that the baseline miRNA expression levels were inconsistently reported across the included studies. In several cases, comparisons with sham or non-stroke controls were not provided, limiting the ability to distinguish disease-related dysregulation from intervention-induced changes. This lack of standardized baseline reporting may affect cross-study comparability and interpretation of therapeutic effects.

Finally, the predominance of unclear risk of bias across multiple domains was largely attributable to incomplete reporting of methodological details in the included studies rather than confirmed methodological flaws. While the included studies met the eligibility criteria for outcome reporting, the lack of transparency in study design and reporting limits confidence in the internal validity and may affect the translational relevance of the findings.

## Conclusion

In conclusion, this review highlights the dual roles of miRNAs in ischemic stroke, as both protective and harmful regulators of injury and recovery. Numerous pieces of evidence indicate that miRNA-based treatments for ischemic stroke are a rapidly advancing, highly promising field for regenerative medicine in neuroscience. Preclinical research consistently demonstrates that miRNAs can simultaneously influence multiple pathological processes, and their regulation can either contribute to ischemic stroke pathology or reduce the ischemic damage and promote recovery. The pleiotropic properties of miRNAs present both advantages and challenges, requiring meticulous design for therapeutic applications. If the challenges of clinical translational research can be overcome, miRNA modulation therapy offers a personalized, multi-mechanistic approach for cerebrovascular disease and, certainly, for other diseases as well.

## Supplemental Information

10.7717/peerj.21571/supp-1 Supplemental Information 1 SYRCLE RoB ratings for each individual study.

10.7717/peerj.21571/supp-2 Supplemental Information 2 Inter-rater reliability between two reviewers for SYRCLE Risk of Bias assessment of included studies.

10.7717/peerj.21571/supp-3 Supplemental Information 3 PRISMA checklist.

## Additional Information and Declarations

### Data Availability

The following information was supplied regarding data availability:

This is a Systematic Review/Meta-analysis.
