# Supplementary material for: A systematic review on the dual roles of microRNAs in ischemic stroke: mechanisms and effects
Source: PeerJ. 2026 Jul 27;14:e21571. doi: 10.7717/peerj.21571 (PMC13421810; doi:10.7717/peerj.21571)
Supplement: Supplemental Information 1 [file peerj-14-21571-s001.docx]

## Table S1. SYRCLE RoB ratings for each individual study

| **Study No** | **Authors** | **Item 1** | **Item 2** | **Item 3** | **Item 4** | **Item 5** | **Item 6** | **Item 7** | **Item 8** | **Item 9** | **Item 10** |
| --- | --- | --- | --- | --- | --- | --- | --- | --- | --- | --- | --- |
| 1 | Deng et al.,2020 | Unclear | Unclear | Unclear | Unclear | Unclear | Unclear | Low | Unclear | Unclear | Low |
| 2 | Pan et al., 2020 | Unclear | Unclear | Unclear | Unclear | Unclear | Unclear | Unclear | Unclear | Unclear | Unclear |
| 3 | Wang et al., 2020 | Low | Unclear | Unclear | Unclear | Unclear | Unclear | Low | Unclear | Unclear | Unclear |
| 4 | Yi, Fang & Li, 2020 | Unclear | Unclear | Unclear | Unclear | Unclear | Unclear | Unclear | Unclear | Unclear | Unclear |
| 5 | Yue et al., 2020 | Unclear | Unclear | Unclear | Unclear | Unclear | Unclear | Unclear | Unclear | Unclear | Unclear |
| 6 | Ghasemloo et al., 2021 | Low | Unclear | Unclear | Unclear | Unclear | Unclear | Low | Unclear | Unclear | High |
| 7 | Lv, Li & Che, 2021 | Unclear | Unclear | Unclear | Unclear | Unclear | Unclear | Unclear | Unclear | Unclear | High |
| 8 | Song W et al., 2021 | Unclear | Unclear | Unclear | Unclear | Unclear | Unclear | Low | Unclear | Unclear | High |
| 9 | Song X et al., 2021 | Unclear | Unclear | Unclear | Unclear | Unclear | Unclear | Low | Unclear | Unclear | Unclear |
| 10 | Wang et al., 2021 | Unclear | Unclear | Unclear | Unclear | Unclear | Unclear | Low | Unclear | Unclear | Low |
| 11 | Xin et al., 2021 | Unclear | Low | Unclear | Unclear | Unclear | Unclear | Low | Unclear | Unclear | Unclear |
| 12 | Yang et al., 2022 | Unclear | Unclear | Unclear | Unclear | Unclear | Unclear | Unclear | Unclear | Unclear | Unclear |
| 13 | Zhu et al., 2024 | Low | Low | Low | Unclear | Low | Low | Low | High | Low | High |
| 14 | Jin et al., 2023 | Unclear | Unclear | Unclear | Unclear | Unclear | Unclear | Low | Unclear | Unclear | Low |
| 15 | Zhu et al., 2021 | Low | Unclear | Unclear | Unclear | Unclear | Unclear | Unclear | Unclear | Unclear | Low |
| 16 | Zhang et al., 2020 | Unclear | Unclear | Unclear | Unclear | Unclear | Unclear | Unclear | Unclear | Unclear | Unclear |
| 17 | Lijuan Huang et al., 2023 | Unclear | Unclear | Unclear | Unclear | Unclear | Unclear | Unclear | Unclear | Unclear | Unclear |
| 18 | Wang et al., 2024 | Low | Low | Unclear | Unclear | Unclear | Unclear | Low | Unclear | Unclear | Unclear |
| 19 | Wang et al., 2020 | Unclear | Unclear | Unclear | Unclear | Unclear | Unclear | Low | Unclear | Unclear | Unclear |
| 20 | Xu et al., 2023 | Low | Low | Unclear | Unclear | Unclear | Unclear | Low | Unclear | Unclear | High |

SYRCLE Items: 1, sequence generation; 2, baseline characteristics; 3, allocation concealment; 4, random housing; 5, blinded animal; 6, random outcome assessment; 7, blinding outcome assessors; 8, incomplete outcome data; 9, selective outcome reporting; 10, other types of bias
