# Supplementary material for: A systematic review on the dual roles of microRNAs in ischemic stroke: mechanisms and effects
Source: PeerJ. 2026 Jul 27;14:e21571. doi: 10.7717/peerj.21571 (PMC13421810; doi:10.7717/peerj.21571)
Supplement: Supplemental Information 2 [file peerj-14-21571-s002.docx]

## Table S2. Inter-rater reliability between two reviewers for SYRCLE Risk of Bias assessment of included studies

|  | Reviewer 2: Unclear (0) | Reviewer 2: Low (1) | Reviewer 2: High (2) |
| --- | --- | --- | --- |
| Reviewer 1: Unclear (0) | 164 | 0 | 0 |
| Reviewer 1: Low (1) | 2 | 24 | 4 |
| Reviewer 1: High (2) | 0 | 2 | 4 |

Total counts: Reviewer 2 (Unclear=166, Low=26, High=8); Total studies = 200

• Observed Agreement (Po): 0.96 (96%)

• Expected Agreement (Pe): 0.701 (70.1%)

• Unweighted Cohen’s Kappa (κ): 0.866

• Weighted Cohen’s Kappa (κ): 0.915

According to the interpretation scale of Landis and Koch (1977), a Kappa value of 0.915 indicates almost perfect agreement between the two reviewers.
